# Agricultural plastic mulch increases pesticide persistence in soils

**DOI:** 10.1002/jeq2.70289

**Published:** 2026-10-08

**Authors:** Claire Coutris, Jenny Klaus, Marit Almvik

**Affiliations:** ^1^ Division of Environment and Natural Resources Norwegian Institute of Bioeconomy Research (NIBIO) Ås Norway; ^2^ Division of Biotechnology and Plant Health Norwegian Institute of Bioeconomy Research (NIBIO) Ås Norway

## Abstract

Plastic mulch films are widely used in agriculture, often in combination with pesticide application. While biodegradable plastic mulch films (BDM) are designed for soil incorporation, their influence on pesticide fate remains poorly understood. We investigated the influence of BDM on the degradation kinetics of four widely used pesticides (boscalid, pyraclostrobin, fluopyram and pyraflufen‐ethyl) in two agricultural soils under controlled conditions. Pesticides were applied at agronomically relevant doses either directly to soil or adsorbed onto a commercially available polybutylene adipate terephthalate‐starch BDM prior to soil incorporation, and pesticides and their relevant metabolites were monitored for 112 days. Adsorption to BDM significantly slowed pesticide degradation for all compounds, increasing degradation half‐lifes (DT50) by factors of 2–26 compared with direct soil application. While degradation pathways were not fundamentally altered, the presence of mulch delayed metabolite formation and prolonged pesticide persistence. Rapid degradation of pyraflufen‐ethyl led to the accumulation of pyraflufen‐ethyl metabolite E‐3, a highly persistent multi‐halogenated metabolite that remains analytically and regulatorily overlooked. BDM itself showed limited mass loss (12%) over the experimental period, suggesting that pesticide‐plastic interactions can persist over environmentally relevant timescales. Overall, this study identifies plastic mulch residues as overlooked modifiers of pesticide persistence and metabolite accumulation in soil, suggesting that plastic‐pesticide interactions warrant further consideration in environmental exposure and risk assessment frameworks.

## Plain Language Summary

Plastic mulch films are widely used in agriculture, often in combination with pesticides. While biodegradable plastic mulch films (BDM) are made for soil incorporation after use, their influence on pesticide fate is poorly understood. We studied how BDM modifies the degradation of four widely used pesticides. Pesticides were applied at agronomically relevant doses either directly to soil or adsorbed onto BDM before soil incorporation. Pesticides and their metabolites were studied over 112 days. BDM strongly slowed pesticide degradation, increasing their half‐life in soil by factors of 2–26. Rapid degradation of the herbicide pyraflufen‐ethyl led to the formation of a highly persistent multi‐halogenated metabolite. BDM itself showed limited mass loss, suggesting that pesticide‐plastic interactions can last for months or longer. Overall, we show that mulch residues increase pesticide persistence in soil and may be considered in environmental risk assessments.

**Abbreviations**
BDM: biodegradable plastic mulch films
DFOP: double first‐order in parallel
FOMC: first‐order multi‐compartment
FTIR: Fourier transform infrared spectroscopy
GLM: general linear model
PBAT: polybutylene adipate terephthalate
PE: polyethylene
PPDB: Pesticide Properties Database
RT: retention time
SFO: single first order

## 1 INTRODUCTION

Biodegradable plastic mulch films (BDM) are applied to agricultural soils to hinder weed growth, reduce evaporation and soil erosion, increase soil temperature, and limit soil contact with crops, thereby contributing to increased food production. Contrary to their conventional counterparts made of polyethylene (PE), which require recovery from agricultural fields after use, the intended end‐of‐life of BDM is direct incorporation into soil by tillage after harvest or at the end of the growing season, where complete transformation of BDM fragments into microbial biomass, water, and CO_2_ is expected within variable timeframes. To fulfill the requirements of the European standard EN 17033 (2018), BDM (or their base materials in pellet or powder form) need to show >90% conversion of their organic carbon into CO_2_ (mineralization) within 2 years under standardized laboratory soil conditions (20°C–28°C). Assessing mineralization of commercial BDM under field conditions is more challenging, but their physical and chemical degradation has been confirmed under various edaphic and climatic conditions (Coutris et al., 2025; Griffin‐LaHue et al., 2022; Hayes et al., 2017; S. Li et al., 2023; Sintim et al., 2020; Y. Yang et al., 2022). However, repeated mulch use and suboptimal degradation conditions, including cold climate or dry soil conditions, have been shown to maintain the presence of BDM fragments in soils beyond their expected lifetime (Coutris et al., 2025; Griffin‐LaHue et al., 2022). Under Nordic field conditions, with yearly mean air temperature of 8°C and soil temperature below 0°C for at least 2 months per year, we previously showed that 52%–93% of the original BDM mass was still present after 2 years, under various physical forms ranging from nearly intact to visibly altered mulch surfaces and microplastics (Coutris et al., 2025). Under a humid continental climate in China, the incorporation of BDM led to the transient formation of macroplastics (>5 mm) and microplastics (0.1–5 mm) in soil: macroplastics were no longer visible after 2.5 years, whereas microplastics peaked in number at 2.5 years and were still present after 3.5 years (S. Li et al., 2023).

While the need for pesticide applications is supposedly reduced by the use of plastic mulch films (both conventional and biodegradable), there is evidence of the concomitant use of pesticides and plastic mulch: Beriot et al. (2023) quantified plastic mulch and pesticide residues in the soil of vegetable farms, where plastic mulch had been used > 5 times in the last 7 years. In conventional farms, plastic mulch had been applied on average twice a year, and there had been 8–12 pesticide applications (covering all three pesticide classes, herbicides, fungicides and insecticides) during the last 18 months. All soils analyzed contained plastic residues (>100 µm), with average concentrations of 2000 particles kg^−1^ and 0.2 g kg^−1^, and more than four pesticide residues, with an average concentration of 0.14 mg kg^−1^. The presence of pesticide residues on plastic mulch films has also been evidenced in farmlands of North China by Guo et al. (2020), who found 20 times higher pesticide concentrations on plastic films (0.09–22.2 mg kg^−1^, including insecticides, herbicides, and fungicides) compared to surrounding soils. While such field studies are very valuable but still scarce, a larger body of evidence from laboratory studies has demonstrated that plastic mulch films act as a sink for pesticide residues, although the extent of sorption varies widely with pesticide hydrophobicity and mulch composition, ranging from limited sorption for more polar compounds to strong sorption for hydrophobic pesticide (Beriot et al., 2020; Brochado et al., 2025; Lan et al., 2021; Y. Li et al., 2024; Nerín et al., 1996; Sahai et al., 2024). In the environment, the main pathway for interaction between mulch films and pesticides is following pesticide application during crop production, another pathway being through adsorption of pesticide residues to mulch debris left after incorporation of BDM into soil or incomplete removal of conventional plastic films. Previous studies reviewed in Campanale et al. (2024) have shown that biodegradable microplastics can strongly interact with pesticides through various mechanisms (e.g., hydrogen bonding and hydrophobic and electrostatic interactions) and act as sorption carriers, which may alter pesticide behavior in environmental systems. However, quantification of pesticide degradation and metabolite formation in agricultural soils as modified by BDM residues is still lacking.

The objective of the present study was therefore to quantify the effect of residues from a commercially available BDM on pesticide degradation kinetics and metabolite formation in agricultural soils, using an agronomically relevant pesticide mixture, application rate, and mulch concentration. Four widely used pesticides with contrasting physicochemical properties were investigated in two agricultural soils, a loam and a silt loam. To specifically test the hypothesis that BDM fragments would modify pesticide degradation kinetics, two agronomically relevant exposure scenarios were used: pesticides were either applied to soil directly or first spread onto BDM before incorporation into soil, and comparable pesticide concentrations were ensured in both exposure scenarios. Degradation kinetics of the pesticides and their relevant metabolites were studied over 112 days under controlled laboratory conditions, following internationally standardized protocols and state‐of‐the‐art analytical approach. The pesticides were (1) boscalid, a broad‐spectrum carboxamide fungicide inhibiting spore germination and germ tube elongation; (2) pyraclostrobin, a broad spectrum strobilurin fungicide inhibiting mitochondrial respiration; (3) fluopyram, a broad spectrum benzamide fungicide inhibiting mitochondrial respiration; and (4) pyraflufen‐ethyl, a phenylpyrazole herbicide inducing cell membrane disruption.

## 2 MATERIALS AND METHODS

### 2.1 Pesticides

An aqueous solution of four pesticides was prepared from three commercial pesticide products: Signum (267 g boscalid active ingredient (a.i.) kg^−1^ and 67 g pyraclostrobin a.i. kg^−1^, BASF AS), Luna Privilege (500 g fluopyram a.i. L^−1^, Bayer AS), and Mizuki (10.6 g pyraflufen‐ethyl a.i. L^−1^, Nichino Europe Co., Ltd.). The concentrations of the four active pesticide substances in the spiking solution were 100 µg mL^−1^ boscalid, 77 µg mL^−1^ fluopyram, 25 µg mL^−1^ pyraclostrobin, and 16 µg mL^−1^ pyrafluflen‐ethyl. The mix solution (500 µL) was spiked to fresh soil or to plastic mulch successively mixed into fresh soil to give 1.67 µg g^−1^ boscalid, 1.28 µg g^−1^ fluopyram, 0.42 µg g^−1^ pyraclostrobin, and 0.27 µg g^−1^ pyraflufen‐ethyl (µg a.i. g^−1^ dry weight [dw] soil). These application rates correspond to the normal area application dose (NAD) in Norway for fluopyram and pyraflufen‐ethyl, and half the NAD for boscalid and pyraclostrobin. Physicochemical properties of the four active substances relevant to their environmental fate in soil are shown in Table 1.

**TABLE 1:** Selected properties of the studied pesticides (Lewis et al., 2016; the Pesticide Properties Database [PPDB], https://sitem.herts.ac.uk/aeru/ppdb/en/index.htm).

	Boscalid	Pyraclostrobin	Fluopyram	Pyraflufen‐ethyl
Pesticide type	Fungicide	Fungicide; plant growth regulator	Fungicide; nematicide	Herbicide
CAS RN	188425‐85‐6	175013‐18‐0	658066‐35‐4	129630‐19‐9
*K*_ow_ (log *K*_ow_) octanol–water partition coefficient Hydrophobicity	912 (2.96) Moderate	9770 (3.99) High	2000 (3.3) High	3090 (3.49) High
*K*_oc_ soil adsorption (mL g^−1^) Mobility	772 Slightly mobile	9315 Non‐mobile	279 Moderately mobile	1949 Slightly mobile
Degradation rate DT_50_ lab 20°C (days) Persistence	484 Very persistent	2.2 Non‐persistent	309 Persistent	0.32 Non‐persistent

Abbreviations: DT50, degradation time for 50%; *K*_oc,_ organic carbon–water partition coefficient.

### 2.2 Biodegradable plastic mulch film

The commercially available biodegradable mulch BioAgri (produced by BioBag/Novamont, purchased at LOG, Norway) used in this study is one of the most frequently encountered in vegetable and berry production in Norway. It is black, unperforated, 12‐µm thick, made of polybutylene adipate terephthalate (PBAT) and starch (in undisclosed proportions). The mulch film is certified as biodegradable according to the European standards EN 17033 (2018) and EN 13432 (2000) and the US standard ASTM D6400‐21 (2021). Physicochemical alterations of the mulch film during the experiment were assessed by recording mass and Fourier transform infrared spectroscopic modifications after 0, 7, 14, 28, 56, 84, 98, and 112 days. BDM pieces were recovered from the soil after the pesticide chemical extraction described in Section 2.5. Recovered BDM pieces were soaked in water for 10 min, ultrasonicated in water for 1 min, rinsed on a 0.5‐mm stainless steel mesh under running water, dried at 20°C for 48 h, and weighed. The chemical composition of the mulch surface (up to a depth of 0.6 µm) was analyzed using a Bruker Tensor 27 FTIR spectrometer in attenuated total reflectance mode, as follows: wavenumber range 4000–650 cm^−1^, resolution 4 cm^−1^, accumulation of 16 scans per measurement, and five mulch pieces per soil type were analyzed for each of the eight timepoints. Processing of the Fourier transform infrared spectroscopy (FTIR) spectral data included a baseline correction within the Bruker OPUS software, export as datapoint table and peak area normalization in R (R Core Team, version R.4.4.1). Peak assignment was done as previously described in Coutris et al. (2025).

### 2.3 Soil

Both soils were collected from the topsoil (0–10 cm) of agricultural fields in Eastern Norway (Apelsvoll, 60.6998 N, 10.8697 E) and Central Norway (Steinkjer, 63.9335 N, 11.4407 E) in 2023 and stored at −20°C until use. Soil characterization performed by Eurofins Agro Testing Norway AS is provided in Table 2, including dry bulk density and volumetric water content determined at the Norwegian University of Life Sciences, Norway. The soil from Apelsvoll was a loam (48% sand, 32% silt, and 20% clay), pH 6.2, with 2.3% total organic carbon (TOC). The soil from Steinkjer was a silt loam (25% sand, 60% silt, and 15% clay), pH 6.0, with 1.6% TOC. To restore soil microbial activity, following OECD Guideline 307 recommendations (OECD, 2025), the soils were thawed for 24 h at 20°C, sieved at <4 mm, and pre‐incubated in the dark at 20°C for 12 days at field capacity (pF2, soil water potential −100 hPa) corresponding to a water content of 25.9% w/w for the loam and 25.0% w/w for the silt loam. During this pre‐incubation, moisture adjustments using MilliQ water were made every 4 days to maintain soil water content at pF2.

**TABLE 2:** Soil properties.

	Soil A	Soil B
$pH_{H_{2}O}$	6.2	6.0
Phosphorus (P‐AL, mg/100 g air dry soil)	7.7 (1.5)	11 (2.1)
Potassium (K‐AL, mg/100 g air dry soil)	8.8 (2.2)	12 (2.9)
Magnesium (Mg‐AL, mg/100 g air dry soil)	9.7 (2.4)	6.4 (1.6)
Calcium (Ca‐AL, mg/100 g air dry soil)	160 (25)	72 (11)
Sodium (Na‐AL, mg/100 g air dry soil)	<1	1.5 (0.29)
Loss on ignition (% dry soil)	5.7	3.5
Total organic carbon (% dry soil)	2.3 (0.1)	1.6 (0.1)
Soil type (texture)	Loam	Silt loam
Sand (0.06–2 mm, % w/w)	48	25
Silt (0.002–0.06 mm, % w/w)	31	60
Clay (<0.002 mm, % w/w)	20	15
Dry bulk density (g/cm^3^)	1.371 (0.002)	1.334 (0.001)
Volumetric water content at −100 hPa (cm^3^/cm^3^)	0.376 (0.001)	0.346 (0.002)

*Note*: Element concentrations correspond the plant available fraction extracted using ammonium lactate (AL). Uncertainty is provided in parentheses

### 2.4 Experimental setup

The experiment featured two agricultural soils, two pesticide treatments (S: pesticides applied directly to soil, M: pesticides applied to plastic mulch before addition to soil), eight timepoints (0, 7, 14, 28, 56, 84, 98, and 112 days), and three replicates per combination of treatments, plus three blanks per soil type without added pesticides, for a total of 102 experimental units. Each experimental unit consisted of a 250‐mL Teflon tube filled with fresh soil equivalent to 30 g dry soil (i.e., 37.8 g fresh loam or 37.5 g fresh silt loam), and either 500 µL aqueous pesticide solution directly applied to the soil surface and homogenized using a glass rod (treatment S), or 60 mg (corresponding to 3750 mm^2^) plastic mulch previously spiked with 500 µL pesticide solution, cut into smaller pieces (≤1 × 1 cm) before addition to soil and mixed into soil with a glass rod, ensuring that all mulch pieces were covered with soil (treatment M). Pesticide addition to plastic mulch was done 1 day prior to incorporation into soil by applying 500 µL pesticide solution as droplets (ca. 5 µL) across the entire mulch surface (3750 mm^2^) and left to dry overnight in the dark at 4°C. The mulch concentration in soil was 0.2% w/w dw (i.e., 2 g kg^−1^), which is within the range of plastic mulch concentrations measured in agricultural soils (Lagos et al., 2025; Nizzetto et al., 2025). Applying the entire dose to the mulch represents an upper‐bound scenario designed to isolate pesticide–mulch interactions. Under field conditions, only part of the dose may contact the mulch, affecting the proportion subjected to mulch‐mediated persistence rather than the underlying mechanism. The tubes with lids not fully closed to ensure aerobic conditions were kept in the dark at 20°C in an incubator cabinet. The soil water content was readjusted to pF2 twice a week using MilliQ water. On sampling days, three replicates per treatment were removed and stored at −20°C until chemical extraction and analysis.

### 2.5 Chemical extraction and analysis

Boscalid and the metabolite boscalid‐hydroxy M510F01 (full names and synonyms of all metabolites are provided in Table S1), fluopyram, pyraclostrobin, pyraflufen‐ethyl, and the metabolite pyraflufen were supplied from Sigma Aldrich, all of PESTANAL analytical standard qualities (>95%). Fluopyram benzamide (99.9%) was purchased from LGC Group. The boscalid metabolite M510F49 (99.9%), pyraclostrobin metabolite BF500‐3 (98.8%), and pyraclostrobin metabolite BF500‐6 (98.8%) were kindly provided by BASF SE. The metabolite fluopyram‐7‐hydroxy (99.7%) was kindly provided by Bayer AG. Stock standards at 1 mg mL^−1^ of each compound were prepared in acetonitrile (ACN). Mixes of parent pesticides (at levels 10–2000 ng mL^−1^) and metabolites (at level 100 ng mL^−1^) were made in ACN and used to prepare calibration standards on the day of the analysis. All standards were stored at −20°C. All solvents were hypergrade (99.9%) and supplied from Supelco.

Chemical extraction of the pesticides and their metabolites was performed on the whole soil samples, directly in the 250‐mL Teflon tubes with fresh soils after 1 h thawing at 20°C using ACN as extraction solvent. Preliminary tests with acetone, methanol, and ACN showed that ACN was the optimal solvent, achieving high extraction efficiencies for all target substances without altering the biodegradable mulch film. Each sample was extracted with 75 mL ACN containing 0.083 µg mL^−1^ deuterated internal standard pyraclostrobin‐N‐methoxy‐d3 (Supelco) for 30 min on a horizontal shaker, followed by centrifugation for 5 min at 1350 × *g*. Extraction was repeated with 50 mL ACN. One mL of the combined supernatant was filtered (0.2 µm Phenex regenerated cellulose syringe filters, Phenomenex) into 2‐mL glass vial (Agilent), diluted 1:1 with ACN:methanol (70:30 v/v), capped, and stored at −20°C until analysis. For quality control, each batch of samples included soil blanks of each soil type (without pesticides nor plastic mulch, in triplicates), spiked control samples, and procedural blanks. No pesticide or metabolite residues were detected in the soil blanks, except fluopyram in the silt loam (6.7 ng g^−1^), a concentration 125‐fold lower than in the spiked silt loam at the end of the incubation and unlikely to affect the degradation kinetics. The extracts were analyzed on a Thermo Fisher Scientific high‐resolution accurate mass Q‐Orbitrap instrument, model Q‐Exactive, coupled to an Ultimate 3000 XRS liquid chromatograph (ultra‐high‐performance liquid chromatography‐high‐resolution mass spectrometry). The analytes were separated on a Thermo Accucore aQ (100 × 2.1 mm i.d., 2.6 µm) column. The mobile phase was (A) water in 5 mM ammonium formiate and 0.1% formic acid and (B) methanol in 5 mM ammonium formiate and 0.1% formic acid. The gradient started at 100% A for 0.5 min, succeeded by a linear gradient to 70% B at 7 min, then to 100% B at 9 min, which was held for 4 min before returning to 100% A. The total run time was 18 min. The injection volume was 1 µL, the LC‐flow was 0.3 mL min^−1^ and the column temperature was 25°C. The analytes were detected as hydrogen‐adducts [M+H]+ after positive electrospray ionization, with analysis in Full MS (mass‐to‐charge ratio [m/z] 100–1100, resolution of 70,000) with data‐dependent MS2 with inclusion list of the targeted compounds to produce product ion spectra (collision energies 20 and 60). Processing of samples was performed using Thermo Fisher Scientific Tracefinder 4.1 and mzVault 2.1. The identification criteria were retention time (RT) matched to the reference standard (±0.1 min), parent ion accurate m/z mass within 5 ppm accuracy, and the presence of at least one targeted fragment ion with accurate mass within 5 ppm accuracy and produced by fragmentation of the parent ion (Table S2). An in‐house library of product ion spectra (MS2) for the pesticides and metabolites built in mzVault aided in the identification. The samples were screened in Full MS for certain metabolites for which there are no commercially available reference standards, that is, the metabolites M510F08 (boscalid metabolite), BF500‐7 (pyraclostrobin metabolite), and pyraflufen‐ethyl metabolites E‐2 and E‐3, but of these, only E‐3 was detected in the soil samples. The E‐3 metabolite was identified by its expected RT range and diagnostic fragments at m/z 213.0226 (C_9_H_7_N_2_OClF, also present in pyraflufen‐ethyl and pyraflufen spectra) and m/z 291.0100 (C_11_H_10_N_2_O_2_Cl_2_F; loss of a CHF_2_). Quantification was based on the peak height of the parent ions of calibration standards solved in soil matrix (1–200 ng mL^−1^), with pyraclostrobin‐d3 (25 ng mL^−1^) as internal standard. Pyraflufen‐ethyl metabolite E‐3 was semi‐quantified using the calibration curve of pyraflufen. Limit of quantification, determined at the level that provided a relative standard deviation < 20% for three replicates and provided fragment ions of the precursor ions, was 1.6 ng g^−1^ for most compounds, 5 ng g^−1^ for pyraflufen‐ethyl, 8 ng g^−1^ for boscalid, and 16 ng g^−1^ for pyraflufen. The recoveries, measured in spiked control samples of both soil types, were in the range 90%–116%. Our laboratory is accredited to International Organization for Standardization/International Electrotechnical Commission (2017) for pesticide analysis and the method performed satisfactorily in annual pesticide screening proficiency tests (Almvik et al., 2023) and in the 2022 proficiency test “Determination of pesticides in agricultural soil” (ÚKZÚZ, Czech Republic; *z*‐scores 0.05–1.12;|z| ≤ 2).

### 2.6 Degradation kinetics and statistical analysis

Degradation kinetics of pesticides (parent compounds and their metabolites, when relevant) were determined using the software CAKE (Computer Assisted Kinetic Evaluation, Version 3.7, Capgemini Engineering 2023). The quantitative results from LC‐HRMS analysis were entered in CAKE as concentrations in ng g^−1^ dw and degradation pathways were adjusted for each compound. Four different kinetic models were tested: single first order (SFO), first‐order multi‐compartment (FOMC), double first‐order in parallel (DFOP), and hockey stick. The modeling outputs were dissipation curves, as well as DT50 and DT90, which, in the present study, represent the time (in days) within which 50% and 90%, respectively, of a substance disappears due to degradation processes. DT50 and DT90 normally characterize dissipation times, encompassing both degradation and transfer processes, but the latter (including volatilization, plant uptake, and run‐off) are not relevant to the present study. Selection of the best model was based on the quality of the fit (lowest *χ* ^2^ value) between the modeled curve and the experimental datapoints, and on the mechanistic plausibility of the model when two models had comparable *χ* ^2^ values. Confidence intervals (95% CI) for model parameters were obtained directly from CAKE. DT50 95% CIs were calculated by lower/upper parameter substitution for most SFO models and by an endpoint profile likelihood approach for FOMC, DFOP, and the SFO models fitted to pyraflufen‐ethyl following direct soil application. For boscalid in soil B following direct soil application, where concentrations on days 0 and 7 were unavailable due to recovery issues, the SFO model was fitted from day 14 onward and the initial concentration back‐extrapolated according to FOCUS guidance (Boesten et al., 2006).

The influence of plastic mulch, soil type, pesticide hydrophobicity (octanol–water partition coefficient [*K*_ow_]) and soil adsorption (organic carbon–water partition coefficient [*K*_oc_]) on the response variable DT50 was analyzed using a general linear model (GLM). Based on residuals plots, the response variable ln(DT50) was natural logarithm transformed before fitting the statistical model. Tukey pairwise comparisons (at 95% confidence) were performed to identify significantly different treatments. Minitab version 21.4 was used for the analysis.

## 3 RESULTS AND DISCUSSION

### 3.1 Pesticide degradation kinetics

The degradation kinetics of boscalid, pyraclostrobin, fluopyram, and pyraflufen‐ethyl in agricultural soils are shown in Figure 1, with numerical data provided in Tables S3 and S4. DT50 values and associated 95% confidence intervals are reported in Table 3, whereas DT90 values and full model outputs are provided in Table S5. While pesticide degradation kinetics in soil are well‐documented, the role of plastic mulch residues as modifiers of degradation has rarely been tested under soil conditions. We will first present and discuss the results from direct pesticide application to soil, including the largely undocumented behavior of relevant metabolites, and then examine how pesticide–plastic sorption altered degradation kinetics.

**FIGURE 1:**
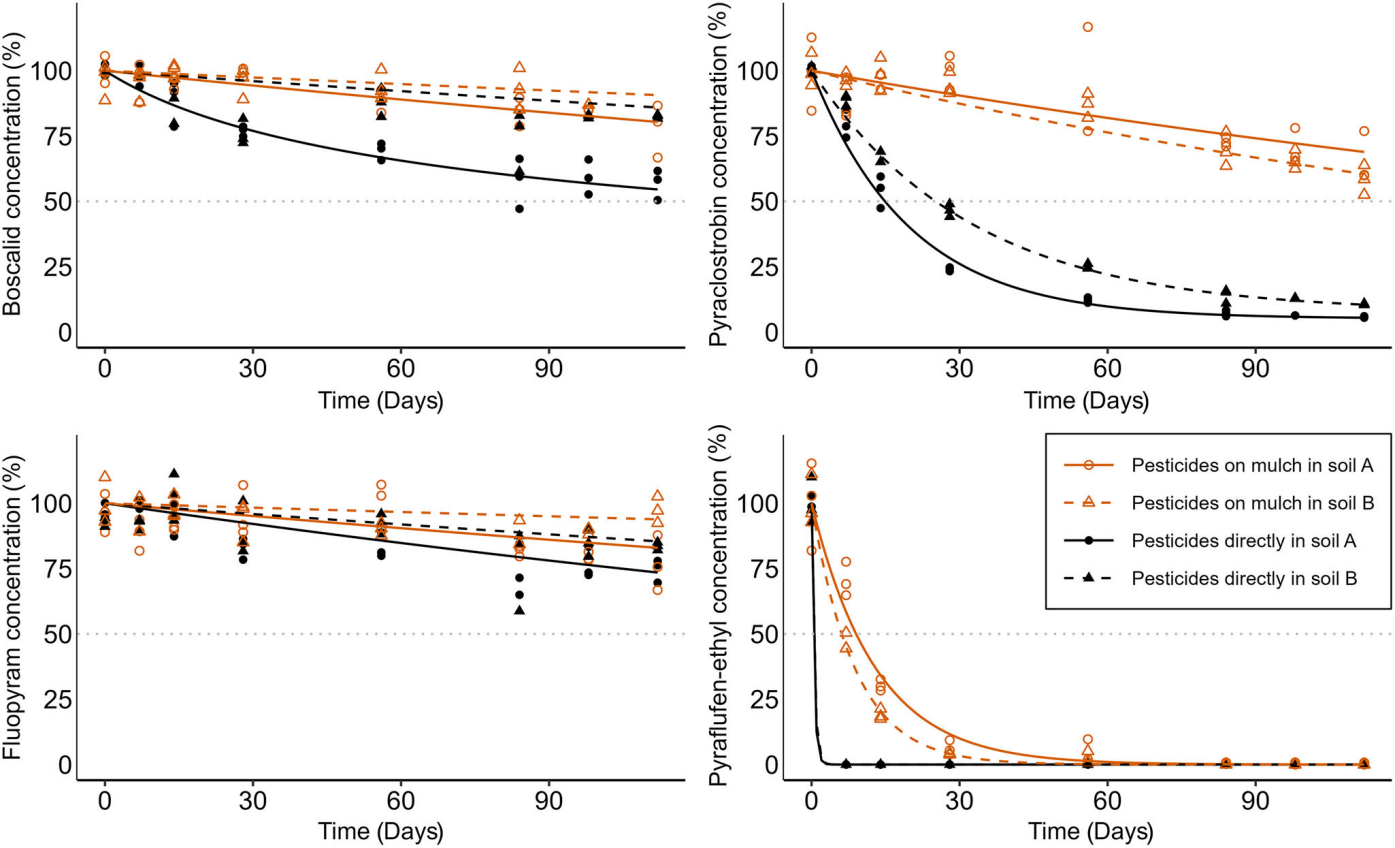
Degradation kinetics of boscalid, pyraclostrobin, fluopyram, and pyraflufen‐ethyl in agricultural soils (soil A: loam; soil B: silt loam) over 112 days (at 20°C, pF2), with pesticides directly added to soil (in black) or adsorbed to plastic mulch before addition to soil (in orange).

**TABLE 3:** Degradation times (at 20°C, pF2) of boscalid, pyraclostrobin, fluopyram, and pyraflufen‐ethyl in agricultural soils (loam and silt loam) when applied directly to soil (treatment soil) or applied to biodegradable plastic mulch before mixing into soil (treatment mulch).

Pesticide	Soil	Treatment	DT50 (days)	95% CI	DT50_Mulch_/DT50_Soil_
Boscalid	Loam	Soil	146	111–225	
		Mulch	356	301–433	**2**
	Silt loam	Soil	510	433–630	
		Mulch	959	815–1155	**2**
Pyraclostrobin	Loam	Soil	15	13–17	
		Mulch	208	139–369	**14**
	Silt loam	Soil	25	23–27	
		Mulch	154	139–186	**6**
Fluopyram	Loam	Soil	252	173–354	
		Mulch	415	231–1009	2
	Silt loam	Soil	493	347–1527	
		Mulch	1220	693–n/a	2
Pyraflufen‐ethyl	Loam	Soil	0.34	0.0–1.0	
		Mulch	9.0	7.6–11.1	**26**
	Silt loam	Soil	0.38	0.0–1.4	
		Mulch	6.1	5.6–6.7	**16**

*Note*: DT50 values represent the time (in days) by which 50% of a compound is degraded and are provided with 95% confidence intervals. n/a: Upper confidence bound not available for highly persistent fluopyram. Ratios given in bold indicate cases where mulch and soil DT50 confidence intervals did not overlap.

Abbreviations: CI, confidence interval.

#### 3.1.1 Pesticides directly applied to soil

When directly applied to soil, the four parent compounds displayed contrasting degradation kinetics (Figure 1) with DT50 ranging from <1 to 511 days (Table 3, treatment S), reflecting their physicochemical properties, and in line with the available literature (Table S6), compiled and regularly updated in the Pesticide Properties Database (PPDB) (Lewis et al., 2016). The degradation of fluopyram and pyraflufen‐ethyl was best fitted to SFO kinetics in both soils. The degradation of boscalid followed SFO kinetics in the silt loam but FOMC kinetics in the loam. Pyraclostrobin displayed DFOP degradation kinetics in both soils. Boscalid and fluopyram were persistent to very persistent in both soils (DT50 146–510 days for boscalid and 252–493 days for fluopyram), while pyraclostrobin and pyraflufen‐ethyl were non‐persistent (DT50 15–25 days for pyraclostrobin and DT50 ≤1 day for pyraflufen‐ethyl). These results, in strong agreement with the published literature compiled in the PPDB, confirm that the experimental setup reproduced expected degradation behavior, providing a robust baseline for assessing plastic mulch‐mediated effects.

Degradation was clearly faster in the loam than in the silt loam for boscalid and pyraclostrobin, as indicated by nonoverlapping 95% confidence intervals, whereas soil‐type differences were less pronounced for the highly persistent fluopyram and negligible for the rapidly degrading pyraflufen‐ethyl (treatment soil in Table 3). While coming from distant geographical regions, both soils are representative of Norwegian agricultural soils, with comparable nutrient status and slightly acidic pH. Their texture differs (loam vs. silt loam), but more importantly, the loam has a higher organic carbon content, albeit less than twofold, than the silt loam. This may have contributed to the observed more rapid degradation in the loam soil, as degradation of pesticides is known to be positively influenced by higher soil organic carbon content due to higher microbial activity (Kah et al., 2007).

In addition to the parent compounds, the concentrations of their relevant metabolites were also determined (Table S4), with maximum molar recoveries reported in Table S7. Of the three boscalid metabolites analyzed, only M510F49 was detected at low and stable levels (8 ± 2 ng g^−1^ dw soil) in both soils throughout the experimental period, in line with the slow and limited degradation of boscalid in soil under aerobic conditions (PPDB; Lewis et al., 2016). In case of fluopyram, the metabolite fluopyram‐benzamide remained below 1.6 ng g^−1^ throughout the experiment. The metabolite fluopyram‐7‐hydroxy was progressively formed over time, reaching 20 ± 2 and 98 ± 10 ng g^−1^ after 112 days in the loam and the silt loam, respectively, with concentrations representing 2% (loam) and 10% (silt loam) of the initial fluopyram concentration (on molar basis). The formation of fluopyram‐7‐hydroxy through hydroxylation of fluopyram is consistent with other findings from aerobic soil incubations in the dark, where fluopyram‐7‐hydroxy was found at maximum 4% of applied radioactivity (AR) from 62 to 121 days (end of the incubation period) (European Food Safety Authority [EFSA], 2013). In case of pyraclostrobin, the metabolites BF500‐3 and BF500‐7 were not detected or were below 1.6 ng g^−1^, while the metabolite BF500‐6 was progressively formed in both soils. In the loam, BF500‐6 reached a maximum of 53 ± 9 ng g^−1^ on day 56, representing 10% (on molar basis, throughout) of the initial pyraclostrobin concentration, and then remained around 8%–9% of the initial pyraclostrobin concentration until day 112. Comparable BF500‐6 concentrations were observed in the silt loam, with a maximum of 59 ± 5 ng g^−1^ on day 112, representing 11% of the initial pyraclostrobin concentration. The metabolite BF500‐6 has been described as a major degradation product of pyraclostrobin under aerobic soil incubation in the dark, found at maximum 31% AR, while the metabolite BF500‐7, not detected in our study, was found at maximum 13% AR, both metabolites presenting moderate to very high persistence (European Food Safety Authority [EFSA] et al., 2025). Given the slow degradation rates of the boscalid, fluopyram, and pyraclostrobin, and the ongoing formation of their metabolites during the experimental period, it was not possible to determine DT50 for the detected metabolites.

In case of pyraflufen‐ethyl, both pyraflufen and pyraflufen‐ethyl metabolite E‐3 were detected (Figure 2, direct application), while pyraflufen‐ethyl metabolite E‐2 was not. Pyraflufen‐ethyl was rapidly transformed into pyraflufen following direct soil application, with high pyraflufen concentrations already detected at time zero. Given the well‐documented very rapid dissipation of pyraflufen‐ethyl in soil (DT50 typically < 1 day; Lewis et al., 2016), the lower than expected time‐zero concentrations likely reflect rapid transformation during the spiking period prior to sample freezing. Pyraflufen was rapidly produced and rapidly degraded, with concentrations ranging from 174 ± 6 to 194 ± 20 ng g^−1^ on day 0 in the loam and the silt loam, respectively, to below 5 ng g^−1^ on day 56 in both soils. The concentration of pyraflufen‐ethyl metabolite E‐3 rapidly increased until day 56 in both soils (reaching estimated levels of 199 ± 19 ng g^−1^ in the loam and 152 ± 4 ng g^−1^ in the silt loam), and slowly decreased afterward, reaching estimated levels of 146 ± 5 and 129 ± 6 ng g^−1^ on day 112 in the loam and silt loam, respectively. Although the herbicide pyraflufen‐ethyl was introduced in 1999, no reference standard is yet commercially available for the persistent multi‐fluorinated and chlorinated pyraflufen‐ethyl metabolite E‐3, hindering its correct quantification in environmental samples. The European Food Safety Authority reported high to very high persistence for pyraflufen‐ethyl metabolite E‐3, with an SFO DT50 of 153–498 days in soil at 20°C and 45% maximum water‐holding capacity (European Food Safety Authority [EFSA], 2015). Despite this, pyraflufen‐ethyl metabolite E‐3 remains largely unaddressed in risk assessments of pyraflufen‐ethyl.

**FIGURE 2:**
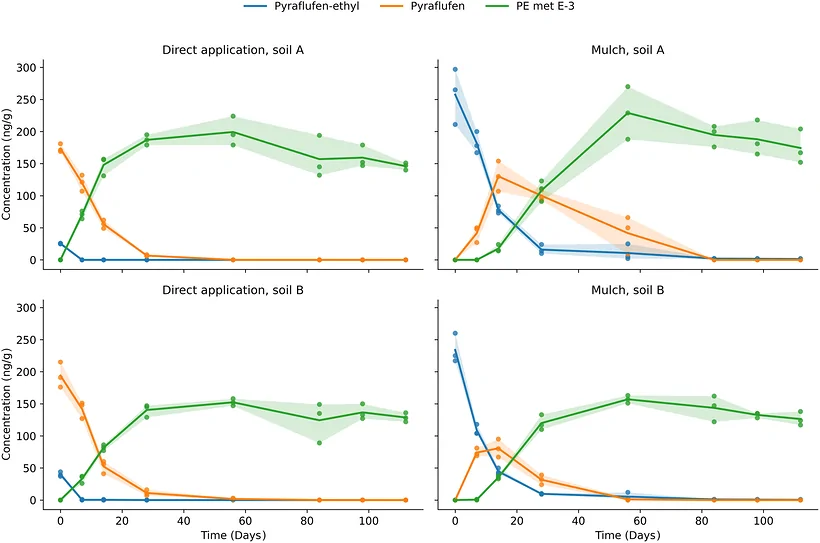
Concentration (in ng g^−1^ dw soil) of the parent compound pyraflufen‐ethyl (in blue) and the metabolites pyraflufen (in orange) and pyraflufen‐ethyl metabolite E‐3 (in green, estimated concentration) over 112 days in soil (at 20°C, pF2), when the parent compound was directly applied to soil or applied to plastic mulch before addition to soil. Soil A: loam, soil B: silt loam.

#### 3.1.2 Pesticides added to soil while adsorbed onto plastic mulch

The degradation of all pesticides in soil was significantly reduced by the fact that pesticides were adsorbed to plastic mulch (GLM, with analysis of variance (ANOVA)/Tukey, *p* = 0.005). This consistent pattern across all pesticides and both soils is consistent with reduced pesticide availability and accessibility to microbial degradation, as expected from sorption of pesticides to the plastic mulch surface. While pesticide–plastic interactions are considered a plausible explanation for the observed effects, alternative explanations involving mulch‐induced changes in microbial activity or community composition cannot be excluded (Lagos et al., 2025). In all cases, degradation was best fitted to SFO kinetics. This systematic shift from FOMC or DFOP kinetics following direct soil application to SFO kinetics in the mulch treatments is consistent with the mulch acting as a relatively homogeneous pesticide reservoir. Release from the mulch may therefore have exerted greater control over the overall degradation rate, reducing the influence of heterogeneous sorption and degradation processes in soil. The degradation half‐life of boscalid and fluopyram was doubled in presence of mulch in both soils, with DT50 up to 959 days (2.6 years) for boscalid and 1220 days (3.3 years) for fluopyram (Table 3). Compared to DT50 obtained with direct pesticide addition to soil, the DT50 of pyraclostrobin was 14 times (loam) and six times (silt loam) longer, and the DT50 of pyraflufen‐ethyl was 26 times (loam) and 16 times (silt loam) longer when adsorbed to plastic mulch in soil (Table 3). DT50 values obtained with mulch were clearly exceeding the range reported in the PPDB (Table S3) for pyraclostrobin and pyraflufen‐ethyl (in both soils) and for fluopyram (silt loam). For boscalid (in both soils) and fluopyram (in loam), the increased DT50 values were still within the range reported in the PPDB. The interaction between either *K*_ow_ (hydrophobicity) or *K*_oc_ (mobility in soil) and the presence of mulch was investigated but did not come out as statistically significant on the sole basis of these four pesticides (GLM, with ANOVA/Tukey, *p* = 0.10). However, we note that the strongest increase in DT50 values, when comparing DT50 with and without mulch, was observed for the two substances with the highest hydrophobicity and soil adsorption capacity, that is, pyraclostrobin (*K*_ow_ 9770, *K*_oc_ 9315) and pyraflufen‐ethyl (*K*_ow_ 3090, *K*_oc_ 1949). In a soil‐free setup including 38 pesticides, Beriot et al. (2020) showed that the sorption of pesticides on several types of agricultural mulch films, including biodegradable ones, was positively correlated with log*K*_ow,_ and that their degradation in water:ACN (90:10 w/w) was significantly reduced in presence of mulch films. In the present study, extraction of the entire soil–mulch matrix did not allow quantitative partitioning of residues between the two compartments. Whole‐system extraction ensured high total residue recovery (90%–116%), but the relative contributions of sorption and mulch‐induced microbial changes could not be determined. Nevertheless, the observed DT50 patterns and previous sorption studies support reduced pesticide availability through sorption as the most plausible explanation.

The formation and degradation of pesticide metabolites over time was determined and all data are compiled in Table S5. For boscalid and fluopyram, the situation was identical to that observed with pesticide applied directly to soil: the boscalid metabolite M510F49 was detected at low and stable levels (8 ± 2 ng g^−1^ dw soil) in both soils throughout the experimental period; fluopyram‐7‐hydroxy was progressively formed over time, reaching 22 ± 2 and 57 ± 4 ng g^−1^ after 112 days in the loam and silt loam, respectively, with concentrations representing 2% and 5% (on molar basis) of the initial fluopyram concentration in the loam and the silt loam, respectively.

In case of pyraclostrobin, the metabolites BF500‐3 and BF500‐7 were not detected or were below 1.6 ng g^−1^, while the metabolite BF500‐6 was progressively formed in both soils. However, the formation of BF500‐6 was much slower in the treatment with plastic mulch than observed with direct pesticide application to soil: it was first detected on day 56 and reached 19 ± 6 ng g^−1^ and 16 ± 6 ng g^−1^ on day 112 in the loam and the silt loam, respectively. The slower formation of BF500‐6 mirrors the increased persistence of the parent compound adsorbed to plastic mulch (Figure 1). In contrast, Lamprou et al. (2025) observed no consistent effect of PBAT‐, starch‐, or low‐density PE‐based microplastics on pyraclostrobin dissipation in soil, suggesting stronger plastic—pesticide interaction for intact mulch films than for microparticles.

In case of pyraflufen‐ethyl, both pyraflufen and pyraflufen‐ethyl metabolite E‐3 were detected (Figure 2, Mulch), while pyraflufen‐ethyl metabolite E‐2 was not. The first metabolite, pyraflufen, went from non‐detectable on day 0 to a maximum of 130 ± 24 and 81 ± 14 ng g^−1^ on day 14 in the loam and the silt loam, respectively. From day 14 onward, pyraflufen concentrations decreased and reached non‐detectable levels on day 56 in the silt loam and on day 84 in the loam. The other metabolite, pyraflufen‐ethyl metabolite E‐3, was first detected on day 14 (estimated to 18 ± 4 ng g^−1^ in the loam and 35 ± 2 ng g^−1^ in the silt loam), reached a maximum on day 56 (estimated to 229 ± 33 ng g^−1^ in the loam and 157 ± 5 ng g^−1^ in the silt loam), and slowly but steadily decreased afterward, with estimated levels of 174 ± 22 and 126 ± 9 ng g^−1^ measured on day 112 in the loam and the silt loam, respectively. As observed with pyraclostrobin, the delayed apparition of pyraflufen‐ethyl metabolites in the treatment with plastic mulch compared to direct soil application is the direct consequence of the slower degradation of the parent compound pyraflufen‐ethyl observed in the treatment with plastic mulch. Modeling of pyraflufen‐ethyl, pyraflufen, and pyraflufen‐ethyl metabolite E‐3 concentrations in CAKE using SFO kinetics indicated that the metabolite pyraflufen had a DT50 of 17 and 7 days in loam and silt loam, respectively, and a DT90 of 56 and 24 days in loam and silt loam, respectively. The degradation of pyraflufen‐ethyl metabolite E‐3 was too slow to be properly modeled. Importantly, pyraflufen‐ethyl metabolite E‐3 accumulates even when pyraflufen‐ethyl is no longer detectable under both pesticide application scenarios, with plastic mulch potentially extending the window of metabolite exposure. This deserves attention given that toxicological data on pyraflufen‐ethyl metabolite E‐3 are still very limited (acute toxicity endpoint 48 h EC50, 50% effect concentration, 0.6 mg L^−1^ for *Daphnia magna*; Olker et al., 2022) and further hindered by the lack of commercially available reference standard.

### 3.2 Biodegradable plastic mulch

The biodegradable plastic mulch had a mass loss of 12 ± 4% after 112 days in soil at 20°C (i.e., 2240 degree‐days), without statistically significant difference between the two soil types (unpaired *t*‐test, *p* = 0.82). This mass loss is in line with what would be expected from the exact same BDM product (BioAgri, 12‐µm thick) after 2240 degree‐days in soil, that is, 10 ± 1% using degradation kinetics derived for BioAgri under field soil conditions (negative quadratic functions; Coutris et al., 2025). Based on these degradation kinetics, >90% mass loss would take 20 months at 20°C and 4 years at 8°C (representative of Nordic conditions). Visual inspection of the plastic mulch under a light microscope showed some limited signs of physical alteration after 112 days, consisting of irregular edges and cracks, compared to the homogeneous‐appearing surface at day 0. FTIR analysis of the plastic mulch also showed some limited spectral changes over time (Figure S1), including (1) a weaker, but broadened peak in band 1 (3600–3100 cm^−1^, O–H stretching) derived from the starch‐associated hydroxyl groups (Smith, 2022), (2) a weakened peak in band 2 (3000–2800 cm^−1^, C–H stretching), (3) an increased peak at wavenumber 1020 cm^−1^ in band 3 (1290–1000 cm^−1^, C–O stretching), which is a pattern of primary alcohols, linked to starch molecules (Smith, 2025), and also reported to increase in the spectra of composted Mater‐Bi by Ruggero et al. (2020). Increased heterogeneity in FTIR spectra of BDM left in soil for 98 and 112 days compared to pristine BDM was observed, indicated by a four‐time increase in the standard deviation of absorbance measurements of soil‐weathered BDM samples compared to their pristine counterparts. This observation supports the assumption that biodegradable plastic materials are not undergoing degradation processes in a spatially uniform manner and that different states of degradation can coexist (Colwell et al., 2023). Finally, the overall limited mulch degradation observed over the course of the experiment suggests that pesticide–plastic interactions can persist over environmentally relevant timescales.

### 3.3 Relevance and limitations under agricultural field conditions

While the controlled incubation conditions used here facilitated mechanistic comparisons among treatments, agricultural soils are considerably more dynamic. Fluctuating temperature and moisture regimes, plant–root interactions, prior weathering of plastic residues, and seasonal shifts in microbial communities may influence pesticide degradation and metabolite formation, potentially altering the magnitude of the effects observed. Nevertheless, controlled laboratory studies remain essential for identifying causal effects of plastic residues on pesticide fate, which may otherwise be difficult to disentangle under field conditions. Although the present study evaluated only a single commercially available PBAT‐starch mulch film, PBAT‐based materials are among the most widely used biodegradable mulches in agriculture. Combined with previous reports of pesticide sorption on various BDM materials, our findings suggest that the observed plastic–pesticide interactions are unlikely to be unique to this product. However, differences in polymer composition, additive content, thickness, crystallinity, and surface properties among BDM films may influence the magnitude of these effects. Future studies should therefore evaluate a broader range of mulch materials to determine the generality and variability of the responses observed here.

Further, the results presented here were obtained from the study of one PBAT‐starch mulch film, which is inherently designed to be incorporated into soil after use; however, some broader implications for conventional plastic mulch films can be considered. Conventional plastic mulch films are generally made of PE and are intended to be removed after use. However, incomplete removal, fragmentation during retrieval, or deliberate incorporation through ploughing and rototillage lead to the accumulation of PE mulch debris, ranging from microplastics to larger fragments, in agricultural soils (Qiang et al., 2023; Steinmetz & Schröder, 2022; X. Yang et al., 2023). The presence of pesticide residues on PE mulch debris has been evidenced (Beriot et al., 2023; Castro et al., 2025; Guo et al., 2020), demonstrating that PE mulch debris can act as vectors of pesticides in the environment. In a comparative sorption study, Sahai et al. (2024) showed that microplastics derived from PBAT mulch films adsorbed a wider range of pesticides compared to microplastics derived from PE mulch films, particularly pesticides with lower *K*_ow_ values. Given the much greater persistence of PE in soil, such interactions could potentially persist for longer periods than those involving BDM residues, although this hypothesis remains to be tested experimentally. Castro et al. (2025) further detected pesticide residues in recycled PE products used in agriculture (with lower pesticide levels in mulch films compared to irrigation pipes and tying tapes), suggesting that pesticides can withstand PE recycling processes. Overall, the available evidence indicates that the combined use of pesticides and plastic mulch, whether biodegradable or conventional, warrants further investigation because of their potential implications for pesticide persistence in the environment.

## 4 CONCLUSION

This study, conducted using a commercially available PBAT‐based BDM, demonstrates that BDM residues can substantially modify pesticide fate in agricultural soils by slowing their degradation. With pesticides adsorbed to BDM prior to soil incorporation, DT50 values were increased by factors of 2–26 compared to direct soil application, consistent with plastic mulch acting as a sink for pesticide residues, limiting their bioavailability to soil microorganisms and delaying transformation processes. The presence of mulch also influenced the formation and persistence of metabolites, notably the accumulation of pyraflufen‐ethyl metabolite E‑3, a highly persistent multi‐halogenated product, which remains insufficiently addressed in regulatory evaluations of pyraflufen‐ethyl. Although BDM are designed to mineralize in soil, degradation times observed under temperate conditions suggest that pesticide–plastic interactions may persist over environmentally relevant timescales. Importantly, the effects described here occurred at mulch concentrations reported in agricultural soils, underscoring their environmental relevance. While our focus was on biodegradable films, the results raise broader questions regarding PE mulch debris, shown to accumulate in agricultural soils due to incomplete removal and fragmentation. Our findings, together with existing evidence of pesticide retention on plastic mulch residues, suggest that both biodegradable and conventional plastic mulch residues are overlooked vectors that can prolong pesticide persistence and promote the accumulation of persistent metabolites in soil. Incorporating the effects of plastic mulch residues on pesticide degradation and metabolite persistence into environmental risk assessments could therefore improve the accuracy of exposure predictions and ensure that long‑term impacts are adequately addressed.

## AUTHOR CONTRIBUTIONS

**Claire Coutris**: Conceptualization; formal analysis; funding acquisition; investigation; resources; validation; visualization; writing—original draft; writing—review and editing. **Jenny Klaus**: Conceptualization; formal analysis; investigation; validation; visualization; writing—original draft; writing—review and editing. **Marit Almvik**: Conceptualization; formal analysis; funding acquisition; investigation; resources; validation; writing—review and editing.

## CONFLICT OF INTEREST STATEMENT

The authors declare no conflicts of interest.

## Supporting information



Supplementary Material

## Data Availability

All numerical data are provided in Supporting Information. Any other data will be made available on request.
