# Supplementary material for: Agricultural plastic mulch increases pesticide persistence in soils
Source: J Environ Qual. 2026 Oct 8;55(5):e70289. doi: 10.1002/jeq2.70289 (PMC13650173; doi:10.1002/jeq2.70289)
Supplement: Supplementary file 1 [file JEQ2-55-0-s001.docx]

**Supplementary Information**

**Agricultural plastic mulch increases pesticide persistence in soils**

Claire Coutris^1*^, Jenny Klaus^1^, Marit Almvik^2^

^1^Norwegian Institute of Bioeconomy Research (NIBIO), Division of Environment and Natural Resources, Ås, Norway

^2^Norwegian Institute of Bioeconomy Research (NIBIO), Division of Biotechnology and Plant Health, Ås, Norway

*****Corresponding author: P.O. Box 115, NO-1431 Ås, Norway; claire.coutris@nibio.no

**Table of contents**

**Table S1.** Full names of purchased/provided metabolites…………………………………………………………..p.2

**Table S2**. Analytical parameters of the compounds on the UHPLC-HRMS-QExactive…………………..p.3

**Table S3.** Pesticide concentrations (in ng/g dry soil) over 112 d in soil (20°C, pF2), when the parent compounds were **directly applied to soil**……………………………………………………………………………………p.4

**Table S4.** Pesticide concentrations (in ng/g dry soil) over 112 d in soil (20°C, pF2), when the parent compounds were **applied to plastic mulch before addition to soil**…………………………………………….p.5

**Table S5.** Model parameters (with 95% confidence intervals) and DT90 values for boscalid, pyraclostrobin, fluopyram, pyraflufen-ethyl and its metabolite pyraflufen…………………………………p.6

**Table S6**. Comparison of DT50 from the present study with those reported in the PPDB……………p.7

**Table S7.** Maximum molar recovery of metabolites, expressed as % of parent transformed into metabolite, on molar basis………………………………………………………………………………………………………….p.8

**Figure S1**. FTIR spectra of biodegradable plastic mulch BioAgri®	……………………………………………….p.9

**Table S1**. Full names (and synonyms when available) of purchased/provided metabolites used in the study.

| **Metabolite** | **Full name** | **Synonym** |
| --- | --- | --- |
| Boscalid-hydroxy M510F01 | 2-chloro-N-(4'-chloro-5-hydroxy[1,1'-biphenyl]-2-yl)nicotinamide | n/a |
| Boscalid M510F49 | N-(4'-chlorobiphenyl-2-yl)-2-hydroxynicotinamide | n/a |
| Fluopyram benzamide | 2-(trifluoromethyl)benzamide | n/a |
| Fluopyram-7-hydroxy | N-(2-(3-chloro-5-(trifluoromethyl)pyridin-2-yl)-2-hydroxyethyl)-2-(trifluoromethyl)benzamide | Fluopyram M08/AE C656948-7-hydroxy |
| Pyraflufen | 2-(2-chloro-5-(4-chloro-5-(difluoromethoxy)-1-methyl-1H-pyrazol-3-yl)-4-fluorophenoxy)acetic acid | Pyraflufen-ethyl metabolite E-1; pyraflufen acid |
| Pyraclostrobin metabolite BF500-3 | methyl-N-[[[1-(4-chlorophenyl)-pyrazol-3-yl]oxy]-o-tolyl]carbamate | M500F007 |
| Pyraclostrobin metabolite BF500-6 | (1-(4-chlorophenyl)-3-[(2-{(Z)-[2-({[1-(4-chlorophenyl)-1H-pyrazol-3-yl]oxy}methyl)phenyl]-ONN-azoxy}phenyl)methoxy]-1H-pyrazole | n/a |

**Table S2**. Analytical parameters for detection and identification of the compounds on the UHPLC-HRMS-QExactive. Analytes marked with an asterisk were screened without a reference standard; of these, only pyraflufen metabolite E-3 was detected. n.d. = not determined.

| Analyte | Sum formula | RT  (min.) | Precursor ion  (quantifier) (m/z) | Fragment ion  (qualifier) (m/z) | Adduct |
| --- | --- | --- | --- | --- | --- |
| Boscalid | C18H12Cl2N2O | 8.45 | 343.03994 | 307.06345 | M+H |
| Pyraclostrobin | C19H18ClN3O4 | 9.38 | 388.10586 | 164.07063 | M+H |
| Fluopyram | C16H11ClF6N2O | 8.74 | 397.05369 | 173.02106 | M+H |
| Pyraflufen-ethyl | C15H13Cl2F3N2O4 | 9.21 | 413.02772 | 338.9909 | M+H |
| **Metabolites:** |  |  |  |  |  |
| Pyraflufen (E-1) | C13H9Cl2F3N2O4 | 8.36 | 384.99642 | 213.02256 | M+H |
| Pyraflufen met. E-2* | C11H7Cl2F3N2O2 | n.d. | 326.99094 | n.d. | M+H |
| Pyraflufen met. E-3* | C12H9Cl2F3N2O2 | 9.32 | 341.00659 | 213.02256  291.0100 | M+H |
| Boscalid-hydroxy M510F01 | C18H12Cl2N2O2 | 7.37 | 359.0349 | 139.9899 | M+H |
| Boscalid met. M510F49 | C18H13ClN2O2 | 8.98 | 325.07383 | 140.0344 | M+H |
| Boscalid met. M510F08* | C18H13ClN2O | n.d. | 309.07892 | n.d. | M+H |
| Pyraclostrobin met. BF500-3 | C18H16ClN3O3 | 9.45 | 358.0953 | 132.0447 | M+H |
| Pyraclostrobin met. BF500-6 | C32H24Cl2N6O3 | 10.91 | 611.13597 | 223.0868 | M+H |
| Pyraclostrobin met. BF500-7* | C32H24Cl2N6O2 | n.d. | 595.14106 | n.d. | M+H |
| Fluopyram benzamide | C8H6F3NO | 5.08 | 190.04743 | 130.0289 | M+H |
| Fluopyram-7-hydroxy | C16H11ClF6N2O2 | 8.08 | 413.0486 | 173.0211 | M+H |
| **Internal standard:** |  |  |  |  |  |
| Pyraclostrobin-D3 | C19D3H15ClN3O4 | 9.46 | 391.12469 | 197.0999 | M+H |

Values below LOQ were in certain cases accepted and reported for some of the metabolites, even though fragmentation had not been performed across the peak; as they provided a good peak of the precursor with the correct m/z and RT, and no interferences were observed in the blank soils.

**Table S3.** Pesticide concentrations (in ng/g dry soil) over 112 d in soil (20°C, pF2), when the parent compounds were **directly applied to soil**. Soil A: loam, soil B: silt loam. Parent compounds: boscalid, fluopyram, pyraclostrobin and pyraflufen-ethyl; metabolites: boscalid M510F49, fluopyram-7-OH, pyraclostrobin BF 500-6, pyraflufen, pyraflufen-ethyl metabolite E-3. *Estimated concentrations for PE metabolite E-3, see Materials and Methods section for further information.

| **Soil** | **Day** | **Boscalid** | **Bosc M510F49** | **Fluopyram** | **Fluop 7OH** | **Pyraclostrobin** | **Pyracl BF 500-6** | **Pyraflufen-ethyl** | **Pyraflufen** | **PE met E-3*** |  |
| --- | --- | --- | --- | --- | --- | --- | --- | --- | --- | --- | --- |
| **Pesticides directly applied to soil - Concentrations in ng/g** | | | | | | | | | | | |
| A | 0 | 798 | 7 | 967 | 0 | 346 | 0 | 26 | 181 | 0 |  |
| A | 0 | 774 | 7 | 957 | 0 | 344 | 0 | 25 | 169 | 0 |  |
| A | 0 | 764 | 8 | 1011 | 0 | 354 | 0 | 25 | 171 | 0 |  |
| A | 7 | 759 | 8 | 989 | 7 | 296 | 21 | 0 | 121 | 76 |  |
| A | 7 | 793 | 7 | 1019 | 6 | 259 | 18 | 0 | 107 | 71 |  |
| A | 7 | 732 | 8 | 1022 | 5 | 274 | 21 | 0 | 132 | 64 |  |
| A | 14 | 741 | 8 | 1044 | 10 | 207 | 50 | 0 | 62 | 156 |  |
| A | 14 | 715 | 8 | 1007 | 9 | 192 | 36 | 0 | 56 | 157 |  |
| A | 14 | 612 | 6 | 882 | 9 | 165 | 38 | 0 | 49 | 131 |  |
| A | 28 | 604 | 6 | 852 | 14 | 81 | 50 | 0 | 6 | 179 |  |
| A | 28 | 583 | 7 | 792 | 12 | 84 | 41 | 0 | 8 | 187 |  |
| A | 28 | 612 | 6 | 872 | 14 | 86 | 48 | 0 | 6 | 195 |  |
| A | 56 | 547 | 8 | 897 | 20 | 46 | 63 | 0 | 0 | 224 |  |
| A | 56 | 561 | 7 | 820 | 20 | 42 | 52 | 0 | 0 | 195 |  |
| A | 56 | 512 | 7 | 807 | 18 | 39 | 46 | 0 | 0 | 179 |  |
| A | 84 | 367 | 5 | 656 | 14 | 21 | 39 | 0 | 0 | 132 |  |
| A | 84 | 463 | 8 | 722 | 20 | 25 | 45 | 0 | 0 | 145 |  |
| A | 84 | 516 | 8 | 854 | 22 | 29 | 57 | 0 | 0 | 194 |  |
| A | 98 | 514 | 8 | 842 | 20 | 22 | 45 | 0 | 0 | 179 |  |
| A | 98 | 459 | 7 | 733 | 17 | 22 | 48 | 0 | 0 | 147 |  |
| A | 98 | 410 | 7 | 742 | 18 | 22 | 50 | 0 | 0 | 152 |  |
| A | 112 | 393 | 7 | 703 | 18 | 20 | 38 | 0 | 0 | 140 |  |
| A | 112 | 454 | 7 | 770 | 20 | 19 | 49 | 0 | 0 | 147 |  |
| A | 112 | 480 | 7 | 787 | 22 | 21 | 47 | 0 | 0 | 151 |  |
| B | 0 | n/a | 9 | 936 | 1 | 326 | 0 | 44 | 191 | 0 |  |
| B | 0 | n/a | 6 | 912 | 1 | 326 | 0 | 39 | 176 | 0 |  |
| B | 0 | n/a | 8 | 946 | 1 | 334 | 0 | 37 | 215 | 0 |  |
| B | 7 | n/a | 10 | 898 | 32 | 284 | 4 | 0 | 127 | 37 |  |
| B | 7 | n/a | 10 | 932 | 28 | 297 | 2 | 0 | 148 | 36 |  |
| B | 7 | n/a | 7 | 936 | 22 | 295 | 6 | 0 | 151 | 26 |  |
| B | 14 | 1496 | 7 | 1113 | 56 | 227 | 14 | 0 | 56 | 77 |  |
| B | 14 | 1023 | 7 | 936 | 46 | 214 | 14 | 0 | 41 | 82 |  |
| B | 14 | 912 | 7 | 955 | 52 | 214 | 15 | 0 | 60 | 86 |  |
| B | 28 | 828 | 6 | 818 | 85 | 145 | 33 | 0 | 10 | 145 |  |
| B | 28 | 846 | 9 | 852 | 96 | 153 | 29 | 0 | 7 | 129 |  |
| B | 28 | 934 | 10 | 1010 | 85 | 161 | 32 | 0 | 16 | 147 |  |
| B | 56 | 942 | 9 | 930 | 100 | 86 | 57 | 0 | 2 | 158 |  |
| B | 56 | 1067 | 10 | 960 | 113 | 80 | 54 | 0 | 0 | 147 |  |
| B | 56 | 1006 | 10 | 883 | 98 | 81 | 49 | 0 | 3 | 152 |  |
| B | 84 | 702 | 9 | 588 | 71 | 36 | 32 | 0 | 0 | 89 |  |
| B | 84 | 947 | 10 | 841 | 107 | 50 | 54 | 0 | 0 | 149 |  |
| B | 84 | 901 | 11 | 876 | 100 | 52 | 54 | 0 | 0 | 135 |  |
| B | 98 | 936 | 11 | 797 | 89 | 42 | 56 | 0 | 0 | 150 |  |
| B | 98 | 946 | 10 | 898 | 89 | 42 | 62 | 0 | 0 | 127 |  |
| B | 98 | 951 | 10 | 849 | 98 | 43 | 59 | 0 | 0 | 133 |  |
| B | 112 | 935 | 13 | 852 | 107 | 36 | 59 | 0 | 0 | 128 |  |
| B | 112 | 946 | 14 | 838 | 99 | 35 | 54 | 0 | 0 | 122 |  |
| B | 112 | 951 | 14 | 822 | 87 | 34 | 64 | 0 | 0 | 136 |  |

**Table S4.** Pesticide concentrations (in ng/g dry soil) over 112 d in soil (20°C, pF2), when the parent compounds were **applied to plastic mulch before addition to soil**. Soil A: loam, soil B: silt loam. Parent compounds: boscalid, fluopyram, pyraclostrobin and pyraflufen-ethyl; metabolites: boscalid M510F49, fluopyram-7-OH, pyraclostrobin BF 500-6, pyraflufen, pyraflufen-ethyl metabolite E-3. *Estimated concentrations for PE metabolite E-3, see Materials and Methods section for further information.

| **Soil** | **Day** | **Boscalid** | **Bosc M510F49** | **Fluopyram** | **Fluop 7OH** | **Pyraclostrobin** | **Pyracl BF 500-6** | **Pyraflufen-ethyl** | **Pyraflufen** | **PE met E-3*** |  |
| --- | --- | --- | --- | --- | --- | --- | --- | --- | --- | --- | --- |
| **Pesticides applied to mulch before addition to soil - Concentrations in ng/g** | | | | | | | | | | | |
| A | 0 | 1138 | 9 | 1305 | 0 | 478 | 0 | 297 | 0 | 0 |  |
| A | 0 | 1026 | 8 | 1121 | 0 | 359 | 0 | 211 | 0 | 0 |  |
| A | 0 | 1068 | 10 | 1218 | 0 | 423 | 0 | 265 | 0 | 0 |  |
| A | 7 | 946 | 7 | 1031 | 1 | 351 | 0 | 178 | 27 | 0 |  |
| A | 7 | 1069 | 9 | 1181 | 3 | 413 | 0 | 200 | 50 | 0 |  |
| A | 7 | 1102 | 7 | 1125 | 3 | 357 | 0 | 167 | 48 | 0 |  |
| A | 14 | 1045 | 8 | 1255 | 5 | 417 | 1 | 73 | 154 | 24 |  |
| A | 14 | 1001 | 7 | 1132 | 5 | 394 | 0 | 84 | 107 | 14 |  |
| A | 14 | 1053 | 8 | 1144 | 5 | 418 | 0 | 77 | 130 | 15 |  |
| A | 28 | 1070 | 8 | 1156 | 11 | 431 | 0 | 10 | 98 | 123 |  |
| A | 28 | 1086 | 10 | 1348 | 11 | 448 | 0 | 24 | 109 | 111 |  |
| A | 28 | 1052 | 7 | 1121 | 10 | 393 | 0 | 14 | 93 | 91 |  |
| A | 56 | 998 | 7 | 1134 | 20 | 326 | 5 | 2 | 9 | 188 |  |
| A | 56 | 904 | 11 | 1350 | 21 | n/a | 14 | 25 | 66 | 270 |  |
| A | 56 | 954 | 8 | 1297 | 16 | 495 | 3 | 5 | 50 | 229 |  |
| A | 84 | 918 | 8 | 1053 | 24 | 307 | 13 | 2 | 0 | 208 |  |
| A | 84 | 965 | 7 | 1041 | 23 | 316 | 6 | 2 | 0 | 176 |  |
| A | 84 | 848 | 7 | 1004 | 22 | 301 | 11 | 2 | 0 | 200 |  |
| A | 98 | 919 | 8 | 1026 | 25 | 331 | 14 | 2 | 0 | 218 |  |
| A | 98 | 916 | 7 | 986 | 19 | 278 | 9 | 0 | 0 | 165 |  |
| A | 98 | 914 | 8 | 992 | 20 | 284 | 19 | 2 | 0 | 181 |  |
| A | 112 | 933 | 9 | 1106 | 24 | 326 | 26 | 2 | 0 | 204 |  |
| A | 112 | 867 | 8 | 953 | 21 | 255 | 17 | 0 | 0 | 167 |  |
| A | 112 | 719 | 7 | 842 | 21 | 254 | 15 | 0 | 0 | 152 |  |
| B | 0 | 1033 | 7 | 1043 | 0 | 365 | 0 | 217 | 0 | 0 |  |
| B | 0 | 1050 | 7 | 1180 | 0 | 395 | 0 | 260 | 0 | 0 |  |
| B | 0 | 920 | 6 | 997 | 0 | 349 | 0 | 225 | 0 | 0 |  |
| B | 7 | 1022 | 8 | 1097 | 15 | 348 | 0 | 104 | 81 | 0 |  |
| B | 7 | 913 | 6 | 957 | 11 | 356 | 0 | 104 | 69 | 0 |  |
| B | 7 | 1011 | 7 | 1074 | 13 | 367 | 0 | 118 | 72 | 0 |  |
| B | 14 | 1048 | 7 | 1028 | 31 | 364 | 0 | 50 | 80 | 35 |  |
| B | 14 | 1007 | 6 | 1021 | 33 | 341 | 0 | 41 | 67 | 33 |  |
| B | 14 | 1058 | 6 | 1109 | 32 | 388 | 0 | 43 | 95 | 38 |  |
| B | 28 | 924 | 6 | 913 | 40 | 338 | 0 | 10 | 24 | 117 |  |
| B | 28 | 1037 | 7 | 1050 | 35 | 343 | 0 | 9 | 39 | 110 |  |
| B | 28 | 1010 | 7 | 1058 | 41 | 368 | 0 | 10 | 32 | 133 |  |
| B | 56 | 956 | 7 | 940 | 52 | 303 | 4 | 2 | 0 | 157 |  |
| B | 56 | 928 | 6 | 991 | 45 | 337 | 4 | 12 | 0 | 151 |  |
| B | 56 | 1042 | 7 | 970 | 57 | 323 | 4 | 2 | 3 | 163 |  |
| B | 84 | 1048 | 8 | 1003 | 64 | 283 | 13 | 0 | 0 | 162 |  |
| B | 84 | 883 | 7 | 905 | 53 | 254 | 14 | 0 | 0 | 147 |  |
| B | 84 | 964 | 12 | n/a | 70 | 235 | 10 | 0 | 0 | 122 |  |
| B | 98 | 902 | 10 | 968 | 59 | 231 | 17 | 0 | 0 | 135 |  |
| B | 98 | 902 | 6 | 945 | 44 | 240 | 14 | 0 | 0 | 135 |  |
| B | 98 | 902 | 16 | n/a | 75 | 258 | 14 | 0 | 0 | 128 |  |
| B | 112 | n/a | 10 | 992 | 58 | 194 | 18 | 0 | 0 | 124 |  |
| B | 112 | n/a | 14 | 1101 | 53 | 236 | 20 | 0 | 0 | 138 |  |
| B | 112 | n/a | 10 | 1043 | 61 | 216 | 9 | 0 | 0 | 117 |  |

**Table S5.** Model parameters (with associated 95% confidence intervals in parentheses) and DT90 values for boscalid, pyraclostrobin, fluopyram, pyraflufen-ethyl and its metabolite pyraflufen in agricultural soils (loam and silt loam), when applied directly to soil (Treatment Soil) or applied to biodegradable plastic mulch before mixing into soil (Treatment Mulch).

| Pesticide | Soil | Treatment | Model | Parameters | DT90 (d) |
| --- | --- | --- | --- | --- | --- |
| Boscalid | Loam | Soil | FOMC | a = 0.4213 (0.07204-0.771)  b =34.84 (18.02-87.69) | 8270 |
|  |  | Mulch | SFO | k = 0.001949 (0.001331-0.003) | 1180 |
|  | Silt loam | Soil | SFO | k = 0.001357 (0.0008472-0.004) | 1700 |
|  |  | Mulch | SFO | k = 8.76E-4 (1.55E-4-0.002) | 3190 |
| Pyraclostrobin | Loam | Soil | DFOP | k1 = 0.05033  k2 = 7.8E-10  g = 0.9488 | 59 |
|  |  | Mulch | SFO | k = 0.003326 (0.00188-0.005) | 691 |
|  | Silt loam | Soil | DFOP | k1 = 0.03096  k2 = 2.36E-12  g = 0.9244 | 117 |
|  |  | Mulch | SFO | k = 0.004495 (0.003726-0.005) | 512 |
| Fluopyram | Loam | Soil | SFO | k = 0.00275 (0.001958-0.004) | 837 |
|  |  | Mulch | SFO | k = 0.001671 (6.87E-4-0.003) | 1380 |
|  | Silt loam | Soil | SFO | k = 0.001407 (4.54E-4-0.002) | 1640 |
|  |  | Mulch | SFO | k = 5.68E-4 (0.0001527-0.001) | 4050 |
| Pyraflufen-ethyl | Loam | Soil | SFO | k = 2.054 (-2055-2.06E+3) | 1.1 |
|  |  | Mulch | SFO | k = 0.07678 (0.06244-0.091) | 30 |
|  | Silt loam | Soil | SFO | k = 1.829 (-1683-1.69E+3) | 4 |
|  |  | Mulch | SFO | k = 0.1136 (0.1034-0.124) | 20 |

| Pyraflufen metabolite | Loam | Mulch | SFO | k = 0.04119 (0.025-0.057) | 56 |
| --- | --- | --- | --- | --- | --- |
|  | Silt loam | Mulch | SFO | k = 0.09605 (0.0775-0.115) | 24 |

SFO: C(t) = C_0_ · exp(-kt)

FOMC: C(t) = C_0_ / (1 + t/b)^a

DFOP: C(t) = C_0_ [g·exp(-k1t) + (1-g)·exp(-k2t)]

**Table S6**. Comparison of DT50 from the present study with those reported in the PPDB (Lewis et al., 2016, <https://sitem.herts.ac.uk/aeru/ppdb/en/index.htm>, accessed 2025). All data are from laboratory studies conducted at 20°C. DT50 values in **bold** are exceeding those reported in the PPDB.

| Pesticide | DT50 (d) | Note | Source |
| --- | --- | --- | --- |
| Boscalid | 484 (103-1214)^1^ | ^1^ Soils = 9 | PPDB |
|  | 146; 511 | Direct soil application | This study |
|  | 356; 959 | Onto mulch in soil | This study |
| Pyraclostrobin | 2^1^ (4 – 95)^2^ | ^1^ Soils = 2 (loamy sand), ^2^ Soils = 7 | PPDB |
|  | 15; 25 | Direct soil application | This study |
|  | **154**; **208** | Onto mulch in soil | This study |
| Fluopyram | 309 (117 – 717) | Soils = n/a | PPDB |
|  | 252; 494 | Direct soil application | This study |
|  | 415; **1220** | Onto mulch in soil | This study |
| Pyraflufen-ethyl | 0.32 (0.2 – 0.4) | Soils = n/a | PPDB |
|  | 0.34; **1** | Direct soil application | This study |
|  | **6**; **9** | Onto mulch in soil | This study |

Lewis, K. A., Tzilivakis, J., Warner, D. J., & Green, A. (2016). An international database for pesticide risk assessments and management. *Human and ecological risk assessment: An International Journal*, *22*(4), 1050-1064. <https://doi.org/10.1080/10807039.2015.1133242>

**Table S7.** Maximum molar recovery of metabolites fluopyram-7-OH, pyraclostrobin metabolite BF 500-6, pyraflufen, and pyraflufen-ethyl metabolite E-3, expressed as **% of parent transformed into metabolite, on molar basis**. The results are based on mean concentrations. The day on which the maximum concentration was observed is indicated in parentheses. For pyraflufen-ethyl directly added to soil, degradation occurred so fast that the reference parent value is the measured parent value at day 0 with mulch.

| Parent | Metabolite | Soil type | Direct soil addition | Applied to mulch first |
| --- | --- | --- | --- | --- |
| Fluopyram | Fluopyram-7-OH | Loam | 2 % (day 112) | 2 % (day 112) |
|  |  | Silt Loam | 10 % (day 112) | 5 % (day 112) |
| Pyraclostrobin | BF 500-6 | Loam | 10 % (day 56) | 3 % (day 112) |
|  |  | Silt Loam | 11 % (day 112) | 3 % (day 112) |
| Pyraflufen-ethyl | Pyraflufen | Loam | 72 % (day 0) | 54 % (day 14) |
|  |  | Silt Loam | 89 % (day 0) | 37 % (day 14) |
| Pyraflufen-ethyl | E-3 metabolite | Loam | 94 % (day 56) | 108 % (day 56) |
|  |  | Silt Loam | 79 % (day 56) | 81 % (day 56) |

**Figure S1**. FTIR spectra of biodegradable plastic mulch BioAgri®, pristine and after various exposure times in soil (at 20°C, pF2), shown as the mean spectrum of 10 samples for each time point. Band 1: O-H stretching (hydroxyl groups), band 2: C-H stretching, band 3: C-O stretching.
